# Clinically suspected acute right ventricular fulminant dengue myocarditis masquerading with dual lethal arrhythmias: a case report

**DOI:** 10.1186/s13256-024-04792-w

**Published:** 2024-11-15

**Authors:** Miftah Pramudyo, Iwan C. S. Putra, Mohammad Iqbal, Hawani S. Prameswari, Giky Karwiky, Triwedya I. Dewi, Pradana Raharjo, William Kamarullah, Norman Sukmadi

**Affiliations:** Department of Cardiology and Vascular Medicine, Faculty of Medicine, University of Padjadjaran, Pasteur Street Number 38, Bandung, Jawa Barat 40161 Indonesia

**Keywords:** Myocarditis, Right ventricle, Complete heart block, Ventricular tachycardia, Case report

## Abstract

**Background:**

Acute right ventricular myocarditis is rare, comprising only 18% of myocarditis cases. Despite being relatively infrequent at 12.4%, dengue-induced myocarditis has a high mortality risk of 26.4%. This report presents a novel case of acute fulminant right ventricular myocarditis due to severe dengue infection, complicated by dual electrical disturbances: complete heart block and ventricular tachycardia.

**Case report:**

A 49-year-old Asian male patient was referred to our hospital with a temporary pacemaker due to a complete heart block. He had a history of recurrent syncope over three days and a fever five days before admission. Initial electrocardiography showed a total atrioventricular nodal block progressing to a high-degree atrioventricular block with a left bundle branch block, indicating an infra-Hisian block. Laboratory findings included thrombocytopenia, elevated troponin, high creatinine, increased liver transaminases, and a positive dengue nonstructural protein 1 test, confirming a diagnosis of dengue infection. Echocardiography showed reduced right ventricular systolic function, normal left ventricular systolic function (ejection fraction: 50%), and dyskinetic intraventricular septum. Coronary angiography revealed normal coronary anatomy. An endomyocardial biopsy was deferred due to severe thrombocytopenia. On the third day, the patient's condition worsened, developing cardiogenic shock and left ventricular systolic dysfunction (ejection fraction: 35%). He subsequently experienced a seizure and slow ventricular tachycardia originating from the right coronary cusp, followed by cardiac arrest. The patient’s family claimed not to resuscitate the patient. Furthermore, the patient died shortly after.

**Conclusion:**

This case underscores the critical need for prompt diagnosis and aggressive management of clinically suspected acute fulminant right ventricular myocarditis because complications can rapidly progress to left ventricular systolic dysfunction, leading to cardiogenic shock and sudden cardiac death.

## Background

Myocarditis is an inflammatory condition of the myocardium triggered by various infectious and noninfectious factors, presenting a broad spectrum of symptoms from asymptomatic to cardiogenic shock, complete heart block, and ventricular arrhythmias [1, 2]. The Global Burden of Disease Study indicates a 62.19% increase in myocarditis incidence from 1990 to 2019, alongside a 65.40% rise in mortality rates [3]. Acute right ventricular (RV) myocarditis is particularly rare, accounting for only 18% of cases, and presents significant diagnostic and management challenges [4]. Viral infections remain the predominant cause of myocarditis. A meta-analysis by Farrukh et al. found that 12.4% of dengue infections result in myocarditis, with a high associated mortality rate of 26.4% [5, 6]. Complications such as high-degree atrioventricular (AV) block and malignant ventricular arrhythmias are even rarer, with incidences of 1.1% and 2.1%, respectively [2]. This report details a rare case of acute fulminant RV myocarditis due to dengue infection, complicated by complete heart block and ventricular tachycardia.

## Case report

**Fig. 1:**
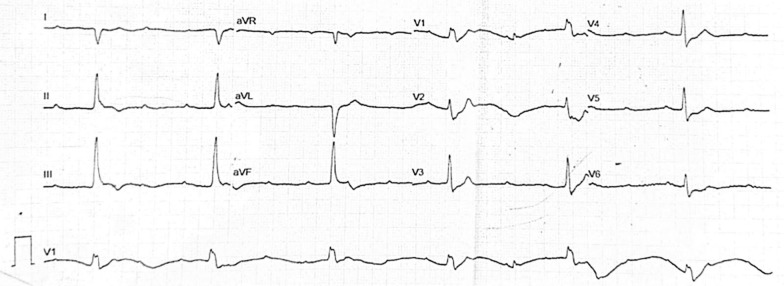
The electrocardiography showed a total atrioventricular nodal block

**Fig. 2:**
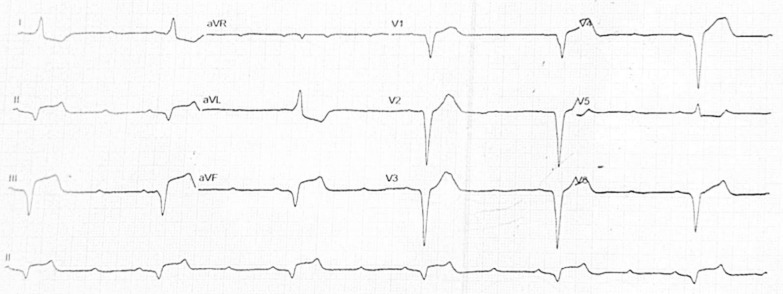
The electrocardiography showed high degree atrioventricular nodal block 3:1 with left bundle branch block

**Fig. 3:**
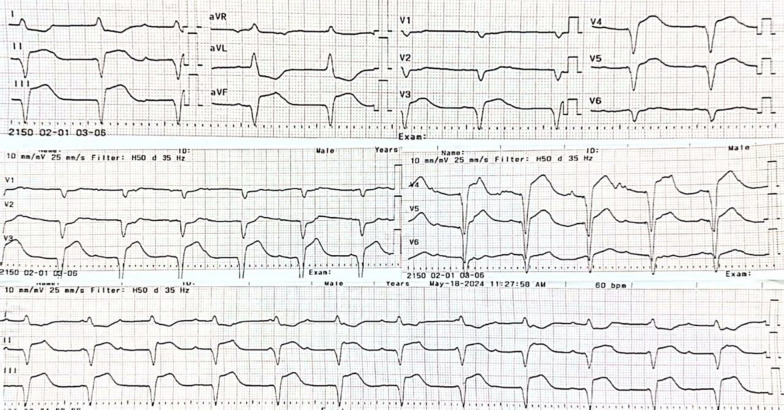
The electrocardiography showed ventricular rhythm pacing with a fixed rate of 60 beats per minute

A 49-year-old Asian male was transferred from a secondary hospital with a temporary transvenous pacemaker set at 60 beats per minute (bpm) due to a complete heart block. He reported 16 syncope episodes over the preceding 3 days, each lasting 10–30 seconds. He also had a fever 5 days prior to admission, accompanied by epigastric pain, nausea, and vomiting. Initial vital signs were stable: blood pressure 110/70 mmHg, heart rate 60 bpm, respiratory rate 20 breaths per minute, temperature 36.7 °C, and oxygen saturation 97% on room air. Physical examination was unremarkable. Electrocardiography from the previous hospital showed a progression from a total AV nodal block (Fig. 1) to a high-degree AV block with a left bundle branch block (LBBB) (Fig. 2), indicating an infra-Hisian block. Moreover, electrocardiography in our hospital showed ventricular rhythm pacing with a fixed rate of 60 beats per minute (Fig. 3).

**Fig. 4:**
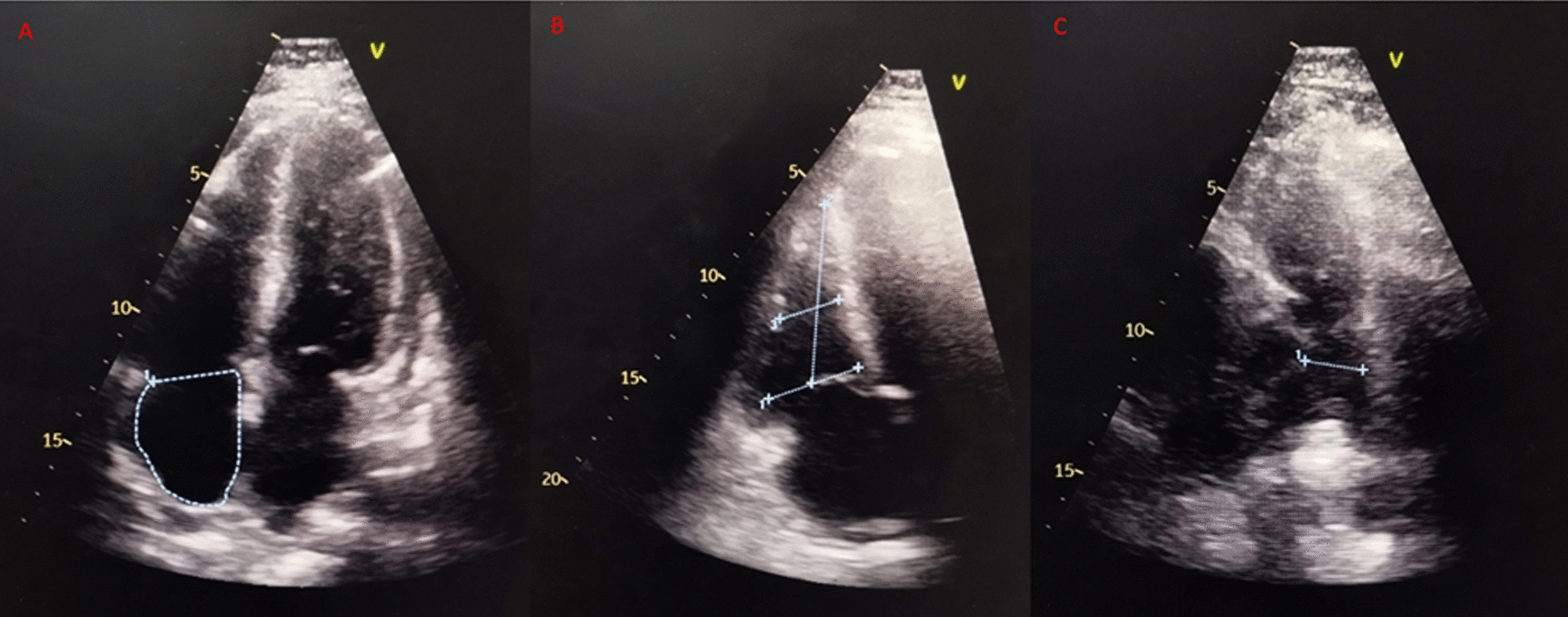
The echocardiography showed (**A**) a right atrium area of 12.5 cm^2^, (**B**) a right ventricle basal diameter of 37 mm, and (**C**) a main pulmonary artery diameter of 19 mm

**Fig. 5:**
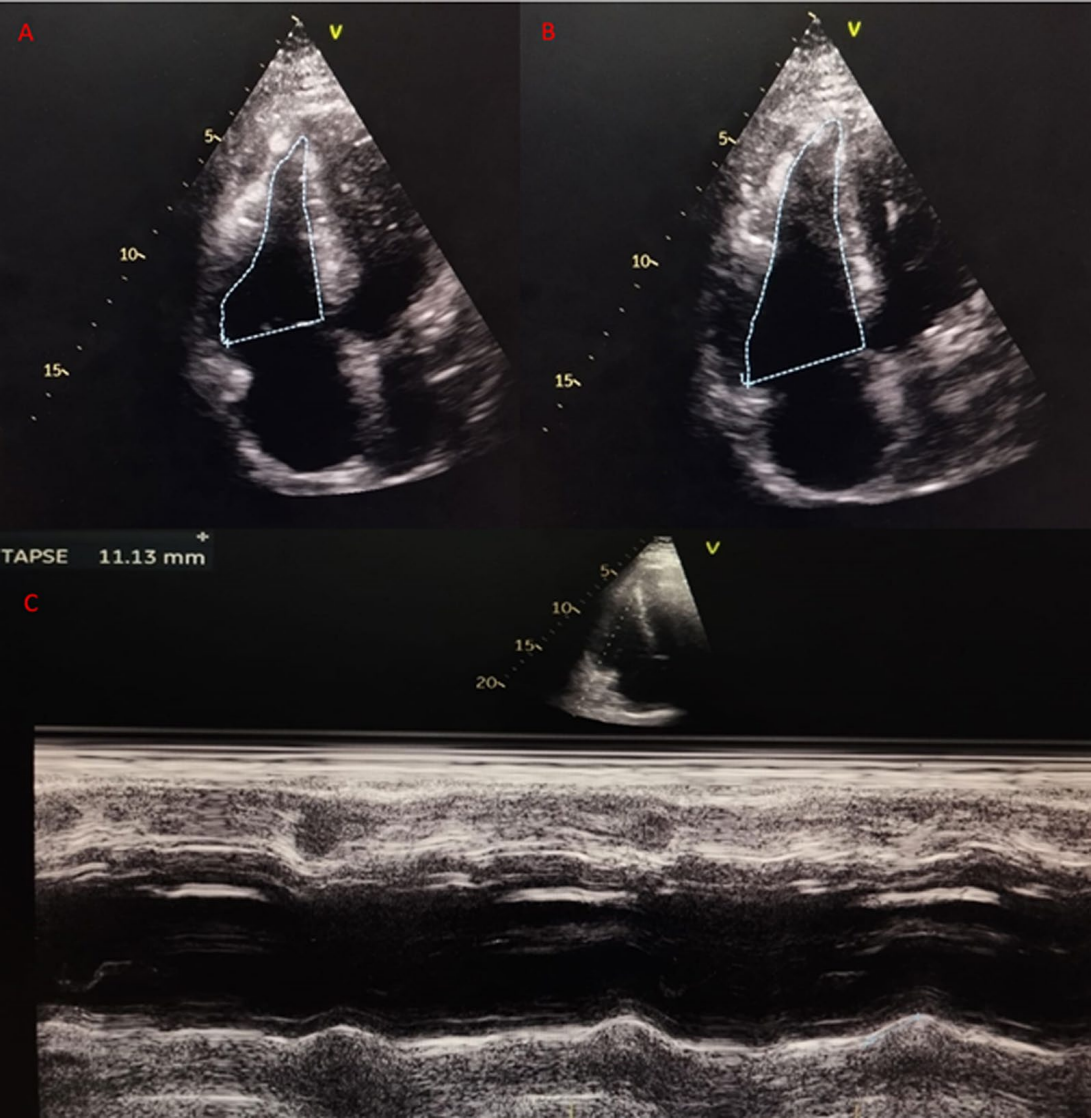
The echocardiography showed (**A**, **B**) fractional change area of the right ventricle of 24% and (**C**) tricuspid annular plane systolic excursion of 11.13 mm

**Fig. 6:**
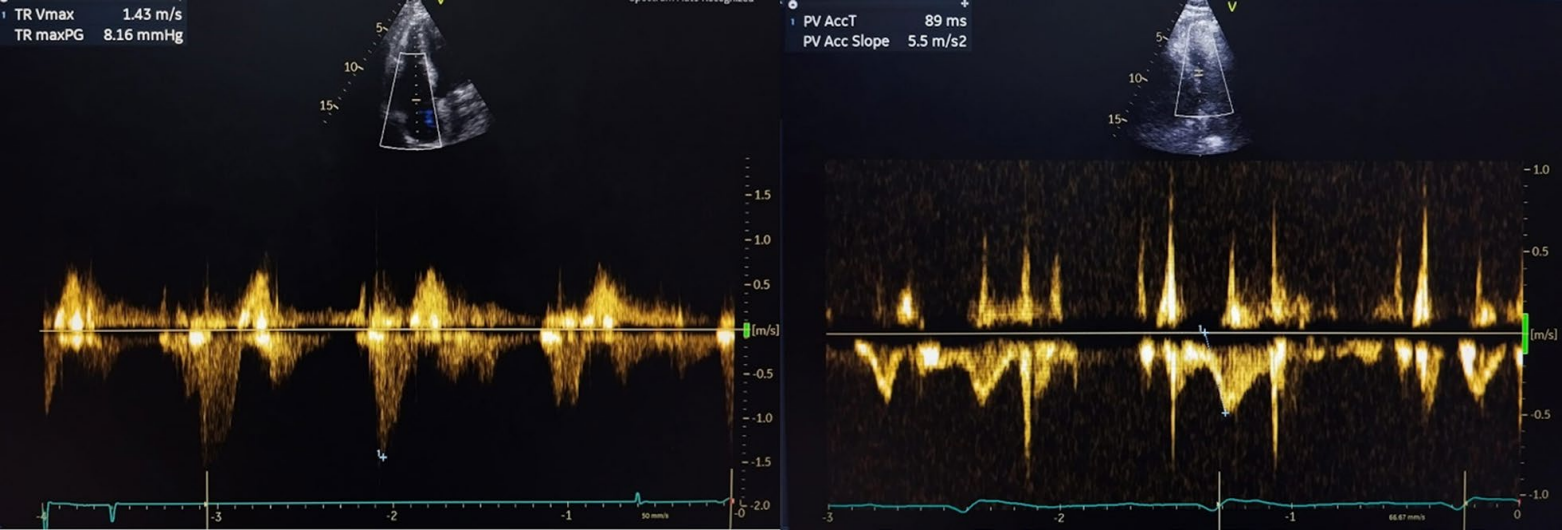
The echocardiography showed tricuspid regurgitation velocity max of 1.43 m/second and pulmonary velocity acceleration time of 89 milliseconds

The chest radiograph showed no evidence of perihilar lymphadenopathy or cardiomegaly. Laboratory findings indicated thrombocytopenia (platelet count 56,000/µL, normal range 150,000–450,000/µL), significantly elevated troponin (10 ng/mL, normal < 0.02 ng/mL), increased creatinine (1.88 mg/dL, normal range 0.72–1.25 mg/dL), and markedly raised liver transaminases (AST: 1806 U/L, normal range 15–27 U/L; ALT: 2276 U/L, normal range 0–55 U/L). The patient also tested positive for dengue nonstructural protein 1 (NS1), confirming the diagnosis of dengue infection. Echocardiography demonstrated normal dimensions of all cardiac chambers, reduced right ventricular (RV) systolic function [tricuspid annular plane systolic excursion (TAPSE): 11 mm, fractional area change (FAC): 22%], normal left ventricular (LV) systolic function [left ventricular ejection fraction (LVEF): 50%], and a dyskinetic intraventricular septum (Figs. 4, 5 and 6). There was mild functional tricuspid regurgitation (TR) and a low probability of pulmonary hypertension. Coronary angiography revealed normal coronary anatomy.

**Fig. 7:**
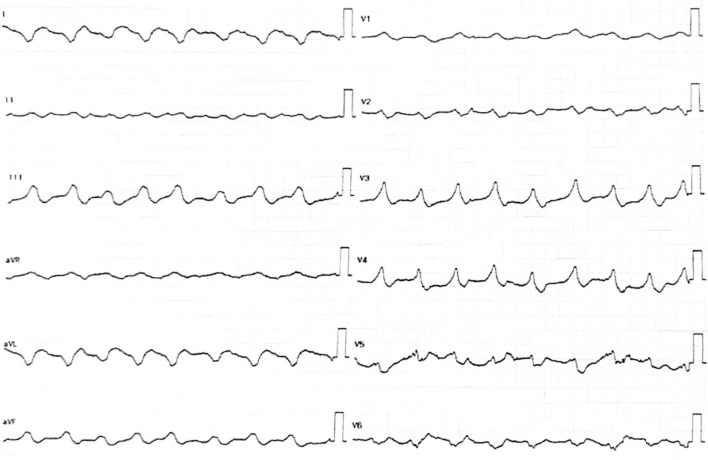
The electrocardiography showed slow ventricular tachycardia

The patient was treated with medications for symptom relief, including lansoprazole 30 mg intravenous twice daily, metoclopramide 10 mg intravenous three times daily, and paracetamol 1000 mg intravenous three times daily. Fluid management was optimized with 2000 cc of Ringer’s lactate over 24 h. An endomyocardial biopsy (EMB) was initially scheduled for the second day of hospitalization but was postponed due to severe thrombocytopenia (platelet count 19,000/µL). On the third day, the patient’s hemodynamic status deteriorated abruptly, evidenced by a drop in blood pressure to 70/40 mmHg, cold extremities, oliguria, and prolonged capillary refill time. Echocardiography showed a decrease in LV systolic function (LVEF 35%), accompanied by a low cardiac index, high systemic vascular resistance, increased estimated pulmonary capillary wedge pressure, distended inferior vena cava, and diffuse bilateral B profile on lung ultrasound, confirming cardiogenic shock. The patient was administered norepinephrine at 0.1 µg/kg/minute and furosemide 80 mg intravenously. Shortly thereafter, the patient experienced a seizure, and electrocardiography (ECG) revealed slow ventricular tachycardia (VT) originating from the right coronary cusp (Fig. 7). Despite immediate interventions, the patient suffered sudden cardiac arrest, and the family requested no resuscitation. The patient subsequently died.

## Discussion

To the best of our knowledge, this is the first reported case of clinically suspected acute fulminant right ventricular (RV) myocarditis due to dengue infection, presenting with dual electrical disturbances: ventricular tachycardia and atrioventricular (AV) block. The AV block episodes included complete heart block and high-degree 3:1 AV nodal block with left bundle branch block (LBBB). Within 3 days of hospitalization, the inflammation extended to the left ventricular (LV) region, reducing LV systolic function and leading to cardiogenic shock and death.

A definite myocarditis diagnosis was not established in this patient due to the inability to perform an endomyocardial biopsy (EMB), attributed to severe thrombocytopenia from the dengue infection. Consequently, according to the European Society of Cardiology guideline, the patient was diagnosed with clinically suspected fulminant myocarditis, meeting one clinical presentation (syncope), three diagnostic criteria (elevated troponin levels, total AV nodal block and ventricular tachycardia on ECG, and new-onset, unexplained reduced RV systolic function, followed by reduced LV systolic function and cardiogenic shock on the third day of hospitalization), and normal coronary anatomy [7]. Other differential diagnoses, such as arrhythmogenic right ventricular cardiomyopathy (ARVC), were excluded as the patient did not fulfill any criteria of the Padua scoring system [8]. Additionally, sarcoidosis was considered unlikely due to the absence of characteristic findings like basal interventricular septum thinning and bilateral hilar lymphadenopathy [9]. Right ventricular infarction was also ruled out due to normal coronary anatomy observed on coronary angiography.

According to current guidelines, platelet transfusion is not advised for thrombocytopenia in dengue infection, regardless of platelet levels, except in cases involving pregnant patients or bleeding [10]. Consequently, we initially refrained from administering platelets to this patient. However, due to their diagnostic importance for treatment decisions, the benefits of platelet concentrate transfusion may outweigh the risks in such patients.

Myocardial involvement in dengue infection can result from direct viral invasion, cytokine-mediated immune responses, or a combination of both. Salgado et al. identified dengue virus (DENV) antigens in various cardiac tissues, including cardiomyocytes, myocardial interstitial cells, and endothelial cells [11]. Research indicates that DEN-2 and DEN-3 serotypes primarily induce myocarditis, highlighting DENV's capacity to invade and injure myocardial cells [12–14]. Dengue infection can also provoke immune-mediated myocardial injury, as pro-inflammatory cytokines and lymphocytes infiltrate myocardial cells, promoting inflammation [5]. However, the pathophysiology of right ventricular predominant myocarditis due to dengue remains unclear.

In this case, systemic congestion due to RV failure was not prominent, likely due to concomitant dengue-induced plasma leakage and resultant hypovolemia. From a pathophysiological standpoint, inflammation and edema may infiltrate the AV node in myocarditis, causing a transient AV nodal block that might resolve as healing progresses. Conversely, infiltration of the His–Purkinje system could lead to permanent scarring and irreversible infra-Hisian block, posing a life-threatening condition [15]. The patient's second ECG showed a high-degree AV nodal block with LBBB, indicating an infra-Hisian block, suggesting the heart block was irreversible. Permanent pacemaker implantation was planned but not performed due to the patient’s death. There are several hypotheses that elucidate the pathophysiology of ventricular arrhythmias in myocarditis. First, the direct cytopathic effect of a viral infection can result in myocardial membrane lysis, creating electrical instability. Second, viral infection can induce endothelial dysfunction, which in turn leads to ischemia-induced ventricular arrhythmias due to coronary microvascular disease. Finally, viral infection may impair the myocardial expression of connexins, leading to gap junction dysfunction and subsequent ventricular arrhythmias [15].

Numerous case reports have documented acute right ventricular myocarditis presenting with complete heart block [16], ventricular tachycardia (VT) [17], cardiogenic shock [18], and combinations of these complications [19]. One such case reported by Sato et al. described a 43-year-old Japanese woman with right ventricular dysfunction caused by lymphocytic myocarditis, which was complicated by VT, complete heart block, cardiogenic shock, right ventricular thrombus, and pulmonary thromboembolism [19]. Similar to our case, her left ventricular systolic function (LVEF 50%) was preserved upon admission but declined abruptly to 20% by the second day of hospitalization, indicating the spread of inflammation to the left ventricular region. Unlike our case, the patient was treated with venoarterial extracorporeal membrane oxygenation (VA-ECMO) to stabilize her hemodynamic condition. The EMB confirmed a diagnosis of lymphocytic myocarditis. Over the course of her hospitalization, her LVEF gradually improved to 59%, although she continued to experience persistent right ventricular dysfunction, complete heart block, and inducible VT. As a result, she received a cardiac resynchronization therapy defibrillator (CRT-D) and was discharged in stable condition on the 56th day of hospitalization.

The use of immunosuppressive therapy in acute viral or lymphocytic fulminant myocarditis remains controversial, in contrast to other forms of fulminant myocarditis such as giant cell myocarditis, eosinophilic myocarditis, and immune checkpoint inhibitor (ICI) myocarditis. Immunosuppression in these cases may risk enhancing viral replication, potentially leading to adverse outcomes [20]. Although the American Heart Association (AHA) recommends considering immunosuppressive treatment for fulminant myocarditis, this guidance is primarily based on case series rather than robust clinical trials [7, 21]. The results of the MYocarditis THerapy with Steroids (MYTHS) trial, which investigates the safety and efficacy of high-dose pulse intravenous corticosteroid therapy for fulminant acute myocarditis, are still pending [22]. Therefore, we initially refrained from administering immunosuppressive therapy to this patient. In alignment with our findings, Sato et al. also withheld immunosuppressive treatment, observing a recovery in left ventricular (LV) systolic function but persistent atrioventricular (AV) nodal block, ventricular tachycardia (VT), and right ventricular (RV) dysfunction [19]. In contrast, a case report by Hama et al. described the use of prednisone at 40 mg/day for a patient with chronic active myocarditis complicated by sustained monomorphic VT, which led to a complete resolution of VT with no recurrences before discharge [17]. Consequently, due to the unpredictable outcomes and the potential for rapid hemodynamic deterioration, immunosuppressive therapy might be advantageous in complex cases of myocarditis. Both our case and that of Sato et al. highlight the necessity of aggressive diagnostic and therapeutic approaches in right-sided fulminant myocarditis, given the potential for irreversible complications and abrupt onset of deteriorating LV systolic function.

A key limitation of this case report is the inability to perform an EMB due to the high risk of bleeding associated with severe thrombocytopenia. Additionally, cardiac magnetic resonance (CMR) imaging was not feasible as the patient was pacing dependent. Consequently, the diagnosis of myocarditis remains inconclusive.

## Conclusion

This case underscores the critical need for prompt diagnosis and aggressive management of clinically suspected acute fulminant RV myocarditis due to dengue infection. The rapid progression to LV systolic dysfunction can lead to cardiogenic shock and sudden cardiac death.

## Data Availability

The data used and/or analyzed during the current study are available from the corresponding author on reasonable request.

## Abbreviations

ALT: Alanine transaminase
AST: Aspartate transaminase
AV: Atrioventricular
bpm: Beats per minute
CMR: Cardiac magnetic resonance
CRT-D: Cardiac resynchronization therapy defibrillator
DEN-2: Dengue virus serotype 2
DEN-3: Dengue virus serotype 3
DENV: Dengue virus
ECG: Electrocardiography
EMB: Endomyocardial biopsy
FAC: Fractional area change
ICI: Immune checkpoint inhibitor
LBBB: Left bundle branch block
LV: Left ventricle/left ventricular
LVEF: Left ventricular ejection fraction
MYTHS: MYocarditis THerapy with Steroids trial
NS1: Nonstructural protein 1
RV: Right ventricle/right ventricular
TAPSE: Tricuspid annular plane systolic excursion
TR: Tricuspid regurgitation
VT: Ventricular tachycardia
